# The emerging role of non-*Helicobacter pylori* microbiome in gastric carcinogenesis: a comprehensive review of pathogenic mechanisms and clinical implications

**DOI:** 10.1093/gastro/goag033

**Published:** 2026-05-21

**Authors:** Minlan Ren, Jing Jia, Yiwei Li, Nanxiang Shen, Yun Shi, Zhe Wang, Xinen Huang, Gang Li, Guanying Xiong, Rui Peng

**Affiliations:** Medical Center for Digestive Diseases, The Second Affiliated Hospital of Nanjing Medical University, 121 Jiangjiayuan, Gulou District, Nanjing, Jiangsu 210000, P. R. China; Department of General Surgery, The Affiliated Cancer Hospital of Nanjing Medical University & Jiangsu Cancer Hospital & Jiangsu Institute of Cancer Research, 42 Baiziting, Xuanwu District, Nanjing, Jiangsu 210009, P. R. China; Medical Center for Digestive Diseases, The Second Affiliated Hospital of Nanjing Medical University, 121 Jiangjiayuan, Gulou District, Nanjing, Jiangsu 210000, P. R. China; Medical Center for Digestive Diseases, The Second Affiliated Hospital of Nanjing Medical University, 121 Jiangjiayuan, Gulou District, Nanjing, Jiangsu 210000, P. R. China; Medical Center for Digestive Diseases, The Second Affiliated Hospital of Nanjing Medical University, 121 Jiangjiayuan, Gulou District, Nanjing, Jiangsu 210000, P. R. China; Department of General Surgery, The Affiliated Cancer Hospital of Nanjing Medical University & Jiangsu Cancer Hospital & Jiangsu Institute of Cancer Research, 42 Baiziting, Xuanwu District, Nanjing, Jiangsu 210009, P. R. China; Department of General Surgery, The Affiliated Cancer Hospital of Nanjing Medical University & Jiangsu Cancer Hospital & Jiangsu Institute of Cancer Research, 42 Baiziting, Xuanwu District, Nanjing, Jiangsu 210009, P. R. China; Department of Medical Oncology, The Affiliated Cancer Hospital of Nanjing Medical University & Jiangsu Cancer Hospital & Jiangsu Institute of Cancer Research, 42 Baiziting, Xuanwu District, Nanjing, Jiangsu 210009, P. R. China; The Fourth Clinical Medical College, Nanjing Medical University, 42 Baiziting, Xuanwu District, Nanjing, Jiangsu 210009, P. R. China; Medical Center for Digestive Diseases, The Second Affiliated Hospital of Nanjing Medical University, 121 Jiangjiayuan, Gulou District, Nanjing, Jiangsu 210000, P. R. China; Department of General Surgery, The Affiliated Cancer Hospital of Nanjing Medical University & Jiangsu Cancer Hospital & Jiangsu Institute of Cancer Research, 42 Baiziting, Xuanwu District, Nanjing, Jiangsu 210009, P. R. China

**Keywords:** non-*Helicobacter pylori*, gastric cancer, historical overview, *Streptococcus anginosus*, *Methylobacterium*

## Abstract

Gastric cancer remains a leading global health concern, with its etiology shaped by complex interactions between the host and its microbiome. The primary etiological role of *Helicobacter pylori* (*H.pylori*) has been well-established, but recent research has pointed to the significant contributions of non*-H.pylori* pathogens in the onset and progression of gastric cancer. These pathogens contribute to gastric tumorigenesis by directly compromising the gastric epithelial barrier and invading gastric epithelial cells, affecting long-range processes, disrupting microbial balance, and influencing the host’s immune microenvironment. In the following, we comprehensively elucidated the potential mechanisms by which *Streptococcus anginosus*, *Methylobacterium*, *Prevotella*, *Candida albicans*, and Epstein-Barr virus actively participate in gastric tumorigenesis. Beyond this, ongoing investigations seek to identify additional microorganisms that may contribute to gastric cancer development, offering new insights into the multifactorial nature of the disease. Collectively, these findings highlight the critical involvement of diverse non*-H.pylori* microorganisms at various stages of gastric cancer progression, advancing our understanding of microbe-driven carcinogenesis.

## Introduction

Gastric cancer was ranked as the fifth most commonly diagnosed malignancy and the fifth leading cause of cancer-related mortality globally [1]. Over the past few decades, *Helicobacter pylori* (*H.pylori*) has gained recognition as a critical pathogenic bacteria responsible for advancing precancerous gastric lesions and leading to the development of gastric cancer. This pathogen employs its unique spiral morphology, urease activity, and the secretion of multiple virulence factors to disrupt the gastric mucosal barrier, facilitating its persistent colonization within the gastric environment [2]. Cytotoxin-associated gene A, a major virulence determinant, enters host cells through the Cag type IV secretion system, triggering a range of intracellular signals and causing chronic inflammation that ultimately promotes the carcinogenesis of the stomach [3]. Vacuum-associated cytotoxin A is another critical virulence factor that contributes to the inflammatory environment by inducing cytokines like interleukin-6 and tumor necrosis factor, disruption of mitochondrial activity, and the induction of programmed cell death [2, 4]. Cytotoxin-associated gene A and vacuum-associated cytotoxin A have been demonstrated to inhibit the growth and antitumor function of CD8+ T cells, which supports immune system evasion [5]. Additionally, *H.pylori* exerts profound effects on the immune microenvironment, including various immune cells, pro-inflammatory cytokines, and chemokines, thereby sustaining chronic gastric mucosal inflammation and accelerating gastric cancer progression [6]. Additional evidence indicates that *H.pylori* influences fibroblast activity, promoting cancer dissemination [7, 8].

The human microbiota constitutes a highly intricate ecosystem. Despite *H.pylori* being present in approximately half of the global population, only 1%–3% of infected individuals eventually progress to malignant gastric tumors [9], implying that other pathogens may also contribute to gastric carcinogenesis. Advances in microbiome research have revealed the contribution of non*-H.pylori* pathogens, whose interactions within the microbial community may further promote cancer. These findings highlight the need to expand research beyond *H.pylori* to understand the broader spectrum of microbial influences on gastric cancer, potentially offering new insights into the disease’s multifactorial nature [10]. In this article, we lay stress on recent advancements in introducing the role of non-*H.pylori* pathogens as causative agents in gastric cancer progression.

## Historical overview of research on non*-Helicobacter pylori* pathogens in gastric cancer

**Figure 1:**
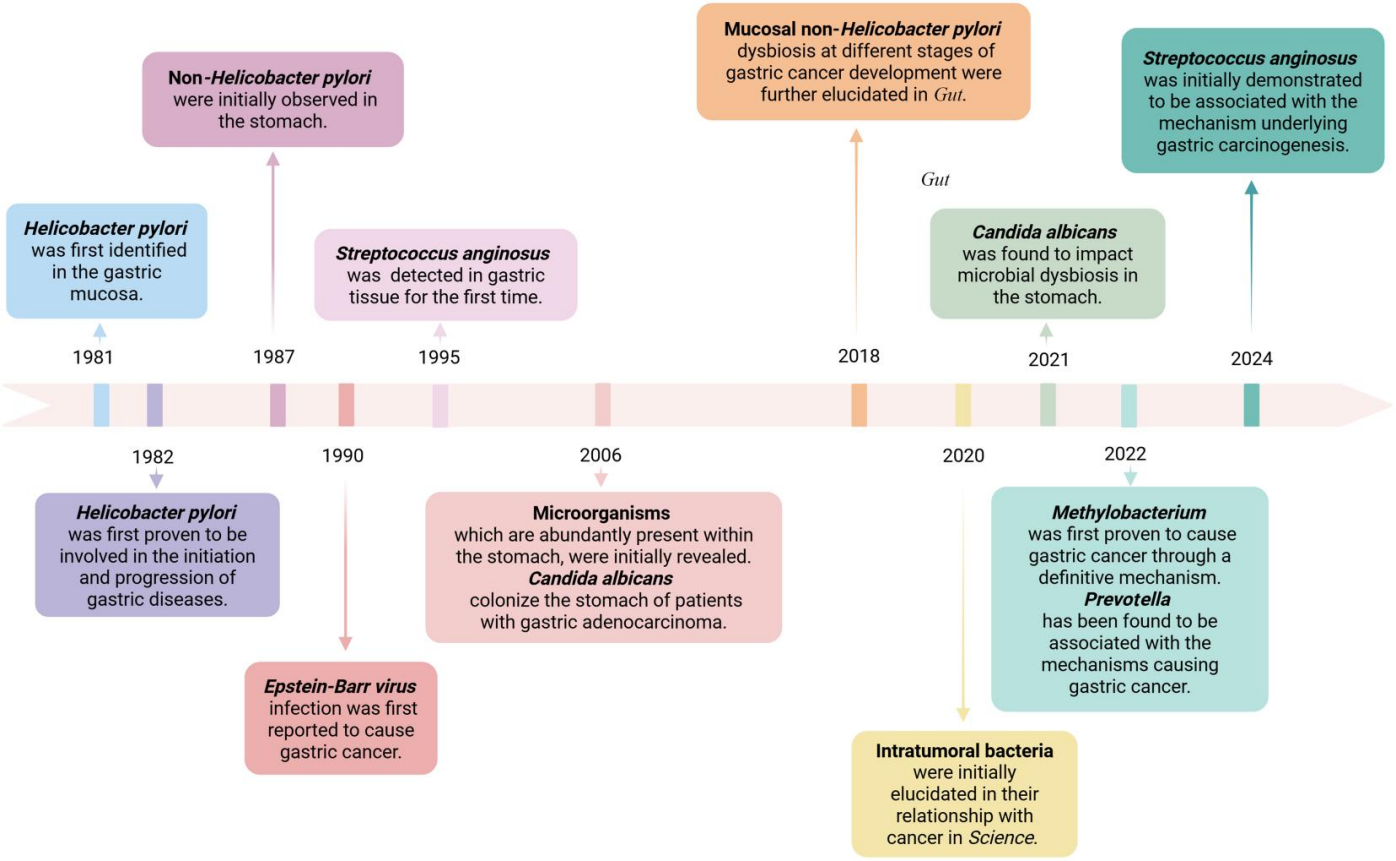
Historical overview of research on non*-Helicobacter pylori* pathogens in gastric cancer. Created with BioRender.com.

*H.pylori* was first identified in 1981, and its definitive association with gastric diseases was established in 1982 [11]. In 1987, Dent *et al.* [12] first observed the presence of non*-H.pylori* microorganisms in the stomach, sparking further investigations into their involvement in gastric disorders such as chronic gastritis, peptic ulcers, and gastric cancer. Despite these observations, the mechanisms by which these microorganisms contribute to gastric diseases remain unclear. Epstein-Barr virus (EBV), for instance, was implicated in gastric cancer in 1990, with later studies showing its association with gastric lymphoepithelioma-like carcinoma and identifying that *EBV* infection accounts for about 6.9% of gastric cancer cases in Japan, with higher prevalence in males and in the upper and middle regions of the stomach [13, 14]. In the following stages, a series of studies further explored the distinct mechanisms through which *Epstein-Barr virus* could contribute to gastric carcinogenesis. In 2006, Bik *et al.* [15] examined gastric biopsy samples from 128 dyspeptic patients, which initially revealed significant microbial diversity in the stomach and identified dominant bacterial phyla such as *Proteobacteria*, *Firmicutes*, *Bacteroidetes*, *Actinobacteria*, and *Fusobacteria*. Later research also confirmed the presence of non*-H.pylori* microbiota within the gastric environment [16, 17]. In 2008, the first experiment using a transgenic mouse model demonstrated that *H.pylori* triple therapy could reverse gastric dysplasia in mice not infected with *H.pylori*, further underscoring the critical role of non*-H.pylori* microorganisms in gastric pathogenesis [18]. Over time, an increasing amount of research has started to examine the role of gastric microbiota in the progression of gastric cancer. Among the most representative studies is the pair of articles published in *Gut* in 2018, which explored the alterations in the microbiota of gastric cancer patients. These pioneering investigations were the first to demonstrate substantial variations in the microbial profiles between individuals with gastric cancer and those with chronic gastritis. Moreover, they uncovered potential genotoxic properties of these microbiota and highlighted the distinct bacterial interactions observed at different stages of gastric carcinogenesis [19, 20]. Although Sasaki *et al.* identified *Streptococcus anginosus* (*S.anginosus*) in gastric cancer as early as 1995, it was not until 2018 that Yu Jun and colleagues conducted an in-depth investigation into five significantly enriched oral microbiota, including *S.anginosus*, within gastric cancer tissues [20]. Subsequently, in February 2024, they confirmed the definitive mechanism by which *S.anginosus* contributes to gastric cancer development [21]. Since *Science* first formally unveiled the relationship between intratumoral bacteria and cancer in 2020 [22], scientists have identified microorganisms such as *H.pylori*, *S.anginosus*, and *Methylobacterium* as intratumoral bacteria influencing the tumor microenvironment of gastric cancer and thereby affecting its onset and progression. Notably, the clear-cut mechanism of gastric carcinogenesis caused by *Methylobacterium* was first discovered by our research group and reported in 2022 [23]. During the same year, scientists also revealed the underlying mechanisms through which *Prevotella* induces gastric cancer [24]. Due to the challenges in culturing fungi and the limitations of existing technologies, although the colonization of *Candida albicans* in the stomachs of gastric adenocarcinoma patients was discovered as early as 2006 [25], only in 2021 did studies reveal a higher abundance of *Candida albicans* in gastric cancer tissues, which was linked to fungal dysbiosis in the gastric microbiome. Nevertheless, the specific pathogenic mechanisms of fungal involvement remain unexplored [26]. Currently, investigations into archaea, fungi, and viruses in gastric cancer are still in their early stages and require further development (Fig. 1).

### The interplay between non-*H.pylori* microorganisms and *H.pylori* in gastric carcinogenesis

Historically, *H.pylori* has been regarded as the predominant causative agent in gastric cancer development. However, emerging evidence suggests that gastric carcinogenesis represents a multi-stage ecological succession process driven by the complex interplay of the gastric microbial community. A growing body of research indicates that non-*H.pylori* microorganisms collaboratively shape the tumor-promoting microenvironment through synergistic, competitive, and dynamically evolving relationships with *H.pylori* [20]. During the initial stages of lesion development, *H.pylori* acts as a keystone species by elevating gastric pH, compromising mucosal integrity, and initiating inflammatory responses, thereby creating a permissive microenvironment for subsequent colonization by non-*H.pylori* species. As gastric pathology progresses to atrophy and intestinal metaplasia, the altered gastric environment becomes less favorable for *H.pylori* survival, leading to a gradual decline in its abundance. Concurrently, non-*H.pylori* microorganisms demonstrate increased relative abundance and assume a more prominent role in driving disease progression. Notably, some studies have revealed that certain species, such as *S.anginosus*, may exert significant carcinogenic effects even during early stages of gastric carcinogenesis. The relationship between *H.pylori* and non-*H.pylori* microorganisms is characterized by dynamic succession rather than static coexistence. At the functional level, these microbial communities establish complementary networks that collectively promote tumorigenesis. Through activation of distinct intracellular signaling pathways, they generate synergistic oncogenic effects, while maintaining tumor-promoting microenvironmental stability through functional redundancy mechanisms. This functional collaboration significantly enhances the overall oncogenic potential of the microbial consortium. In the realm of immune regulation, these microbial populations hierarchically reshape the tumor immune microenvironment. While *H.pylori* establishes a fundamental inflammatory framework, non-*H.pylori* microorganisms further augment immunosuppressive states through multiple mechanisms, collectively leading to functional impairment of anti-tumor immune responses and facilitating the transition from immune activation to immune escape [27]. Furthermore, non-*H.pylori* microorganisms play a particularly crucial role in *H.pylori*-negative gastric cancer, a distinct clinical subtype characterized by unique microbial profiles [28]. In these cases, gastric carcinogenesis appears to be directly driven by specific non-*H.pylori* microbial communities. In conclusion, the relationship between *H.pylori* and non-*H.pylori* microorganisms extends beyond simple synergy, resembling rather a well-orchestrated relay that collectively drives the initiation and progression of gastric cancer.

## The role of non*-H.pylori* pathogens in distinct phases of gastric cancer development

**Figure 2:**
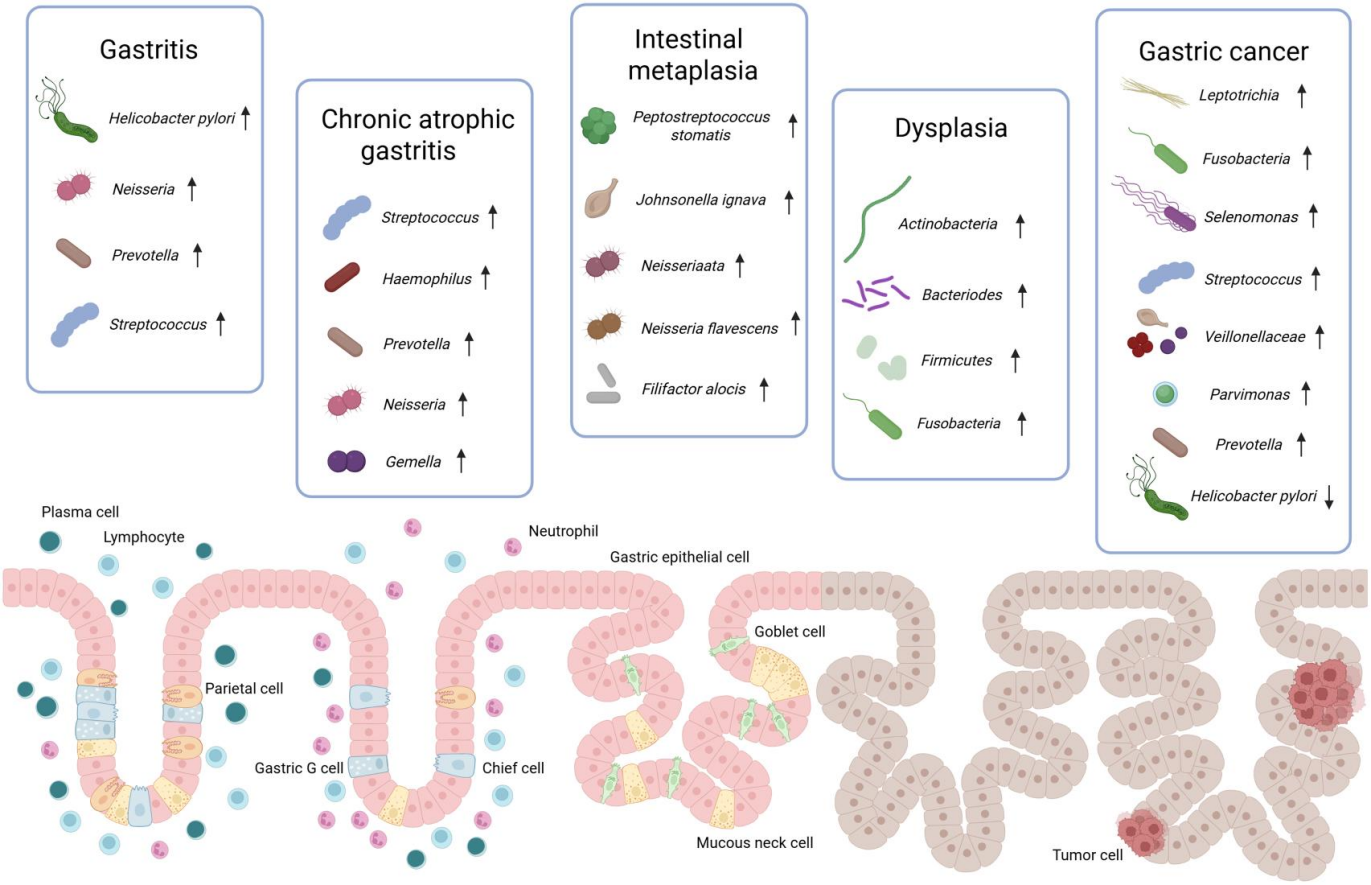
The role of non-*Helicobacter pylori* pathogens in distinct phases of gastric cancer development. Created with BioRender.com.

Gastric cancer generally arises following the progression of various precancerous conditions and lesions. Histopathological analysis classifies gastric cancer progression into distinct stages: chronic gastritis (including non-atrophic and atrophic), intestinal metaplasia, dysplasia (ranging from low- to high-grade), and eventually gastric cancer [29, 30]. Among the many risk factors, *H.pylori* infection remains a key contributor, causing gastric mucosal damage, inflammation, and histological changes. However, emerging studies examining the gastric microbiota have revealed notable differences in microbial community composition at each stage of gastric cancer progression. These findings emphasize the distinct roles that non*-H.pylori* bacteria play in the in gastric carcinogenesis. Due to the heterogeneity of gastric microbiota, variations may exist across populations and between different regions of the stomach. Research has shown that the healthy human stomach is predominantly populated by five major phyla: Proteobacteria, Firmicutes, Bacteroidetes, Actinobacteria, and Fusobacteria [31]. Ferreira *et al.* [19] performed 16S rRNA gene sequencing on tissue samples from 81 patients with chronic gastritis and identified significant enrichment of *Helicobacter*, *Neisseria*, *Prevotella*, and *Streptococcus*. In contrast, in 22 *H.pylori*-negative patients with autoimmune atrophic gastritis, *Streptococcus* emerged as the dominant genus, followed by *Haemophilus*, *Prevotella*, *Neisseria*, and *Gemella* [32]. Wu *et al.* analyzed 89 samples of gastric intestinal metaplasia and observed elevated abundances of *Peptostreptococcus stomatis*, *Johnsonella ignava*, *Neisseria elongata*, and *Neisseria flavescens* [33]. Similarly, Wang *et al.* [34] investigated the gastric microbiota of 25 patients with gastric mucosal dysplasia (including both low- and high-grade dysplasia) and found significant enrichment of *Actinobacteria*, *Bacteroidetes*, *Firmicutes* and *Fusobacteria*. In gastric cancer patients, a decline in the abundance of *H.pylori* has been consistently noted [35]. A meta-analysis involving 2,198 patients with gastric diseases revealed that gastric cancer tissues are enriched with *Leptotrichia*, *Fusobacteria*, *Selenomonas*, *Streptococcus anginosus*, *Veillonellaceae*, *Parvimonas*, and *Prevotella* [36]. Coker *et al.* [20] also identified that *Peptostreptococcus stomatis*, *S.anginosus*, *Parvimonas micra*, *Slackia exigua*, and *Dialister pneumosintes* exhibit centrality within the gastric cancer ecological network. These findings suggest that non*-H.pylori* bacteria serve as persistent stimulatory factors promoting gastric carcinogenesis. Additionally, it has been reported that the relative abundance of *Slackia*, *Bergeyella*, *Selenomonas*, and *Capnocytophaga* increases progressively during the Correa cascade, indicating that distinct oral microbial compositions may serve as biomarkers for differentiating various stages of the Correa cascade [30]. Collectively, the microbial shifts observed at each stage of gastric lesions highlight the significant influence of non*-H.pylori* bacteria in driving gastric cancer progression (Fig. 2).

## Representative non-*H.pylori* pathogenic microorganisms in gastric cancer

### Bacteria

#### Streptococcus anginosus

*S.anginosus* is a Gram-positive coccus commonly found as a commensal organism in the human oral cavity, nasopharynx, gastrointestinal tract, and vaginal tract [37]. Under conditions of immunosuppression, *S.anginosus* can act as an opportunistic pathogen, invading the bloodstream and promoting various diseases. Numerous studies have demonstrated that *S.anginosus* contributes to abscess formation [38–40], purulent inflammation, coronary atherosclerosis [41], urinary and genital tract infection [42], and certain malignancies. Additionally, a few studies have suggested a potential association between *S.anginosus* and central nervous system infections [43].

*S.anginosus* has long been implicated in the pathogenesis of gastric cancer, with studies dating back to 1995 identifying its presence in gastric cancer tissues [44], but further research was limited due to technical constraints. More recently, numerous studies have confirmed the significant enrichment of *S.anginosus* in precancerous lesions and gastric cancer tissues [20, 36, 45–47]. A landmark study published in *Cell* by Fu *et al.* [21] utilized a mouse model to explore the mechanisms by which *S.anginosus* contributes to gastric cancer. Using a mouse model infected with *S.anginosus*, they demonstrated that the bacterium colonizes gastric epithelial cells, inducing both acute and chronic inflammation through the production of neutrophil-derived chemokines like Ccl20 and Ccl8. Over time, *S.anginosus* was shown to drive the progression of gastric lesions from atrophic gastritis and intestinal metaplasia to dysplasia. By the ninth month of infection, its pathogenic effects were found to rival those of *H.pylori*. Further experimentation using YTN16 allograft and MNU-induced gastric cancer mouse models revealed that *S.anginosus* accelerates tumor development. Moreover, in *S.anginosus*-infected mice, the tight junctions between gastric epithelial cells were disrupted. Using 16S rRNA sequencing to analyze the gastric microbiota, they observed that *S.anginosus* altered gastric microbial homeostasis, decreasing beneficial probiotics like *Bifidobacterium* and *Lactobacillus*, while increasing oral commensals, thus contributing to microbial dysbiosis. To further investigate the colonization sites and pathogenic mechanisms of *S.anginosus* in the stomach, the team employed pull-down assays and co-immunoprecipitation techniques to identify specific protein interactions between GES-1/AGS cells and *S.anginosus*. They discovered that *S.anginosus* adheres to gastric epithelial cells through the interaction of its surface protein TMPC with the Annexin A2 receptor. This interaction facilitates long-term colonization of the gastric mucosa and activates several oncogenic pathways, particularly the MAPK/ERK/JNK signaling pathway, promoting gastric cancer progression [21]. In addition, Yuan used 16S rRNA sequencing and unsupervised clustering on tumor tissues and adjacent non-tumor tissues collected from clinical samples, classified tumor samples into six clusters based on 30 highly abundant genera [48]. They observed that patients with tumors enriched in *Pseudomonas* or *Streptococcus* had lower overall survival rates compared with other clusters. Through CIBERSORT, immunohistochemistry, and fluorescence *in situ* hybridization (FISH), the study revealed that *S.anginosus* suppresses antitumor CD8+ T cells and increases tolerance/immune-evading M2 macrophages within tumors. These findings were further validated in spontaneous gastric cancer mouse models, tumor xenograft mouse models, and *in vitro* co-culture experiments with gastric cancer cells, showing that *S.anginosus* and its metabolites promote gastric cancer cell proliferation, migration, and invasion, effects that were attenuated by antibiotic treatment. Metabolomics analysis and KEGG pathway mapping of amino acid metabolism further demonstrated that *S.anginosus* impacts the tumor immune microenvironment by metabolizing arginine into ornithine, thereby promoting gastric cancer progression [48]. Senthil Kumar *et al.* further discovered that *S.anginosus* may activate macrophages by altering cell morphology rather than viability, inducing IκBα phosphorylation and thereby activating the NF-κB transcription factor. This activation promotes the production of pro-inflammatory cytokines (e.g. tumor necrosis factor-α, interleukin-6, and interleukin-1β), inflammatory mediators (e.g. inducible nitric oxide synthase and cyclooxygenase-2), upregulates aconitate decarboxylase expression, increases extracellular acidification rates, and modulates the immunoregulatory metabolic profile of macrophages, ultimately influencing gastric cancer progression [49]. Additionally, other studies have found that in patients with autoimmune atrophic gastritis, the gastric microbiota is predominantly composed of *Streptococcus* species, with higher microbial diversity compared with atrophic gastritis caused by *H.pylori* [50]. These findings underscore the multifaceted role of *S.anginosus* in gastric carcinogenesis, from microbial dysbiosis to immune modulation and oncogenic signaling, emphasizing its significance as a driver of gastric cancer development.

#### Methylobacterium

The genus *Methylobacterium* comprises aerobic or facultatively anaerobic Peptostreptococcus stomatis Gram-negative bacilli with methylotrophic and methanotrophic properties. These bacteria are commonly found in water sources, plants, soil, and hospital environments and typically do not directly cause human diseases [51]. However, in immunocompromised individuals or those with prolonged exposure to specific environments, *Methylobacterium* can lead to disease. Due to its ability to grow in drinking water, adhere to the surface of piping materials, and withstand high temperatures, infections are often associated with patients exposed to contaminated water sources or indwelling medical devices [52]. Studies have confirmed that *Methylobacterium* can cause bacteremia [53], peritonitis [54], infectious endocarditis [55], malignant pleural effusion [56], as well as various cancers, including breast cancer [57, 58], gastric cancer [23], and bladder cancer [59].

A study by Peng *et al.* [23] provided significant insights into the relationship between the gastric microbiota and gastric cancer, highlighting the role of *Methylobacterium* as a key bacterium associated with worse patient outcomes. Through 16S rRNA sequencing and diversity analyses, the researchers compared the gastric and fecal microbiota between gastric cancer patients and those with chronic gastritis. While they found significant differences in gastric microbiota composition, they could not establish a definitive connection between intratumoral bacteria and the fecal microbiota. *Methylobacterium* showed significant enrichment in gastric tumors, with survival analyses indicating that its presence was associated with a worse prognosis, particularly through its involvement in vascular cancer thrombosis. To investigate further how *Methylobacterium* influences the progression of gastric cancer, the study employed techniques including immunohistochemistry, scRNA-seq, flow cytometry, and RT-PCR. They found that *Methylobacterium* within gastric tumors suppresses tumor-resident memory CD8+ T cells (TRM), reduces TGF-β expression, and promotes the formation of tumor-associated vascular emboli. These effects were confirmed in an orthotopic gastric cancer mouse model, where the bacterial infection led to larger tumors and a significant reduction in CD8 + TRM cells within the tumor microenvironment, further supporting the bacterium’s role in promoting tumor progression and contributing to poor prognosis. Additionally, Yang discovered that certain microbes, including *Methylobacterium-Methylorubrum*, showed a significant increase in *Methylobacterium* abundance within distal gastric cancer tissues compared to surrounding normal tissues [60]. Metabolomic analyses revealed alterations in several metabolites, such as adrenic acid, L-pyroglutamic acid, acetyl phosphate, and D-glutamine, while reducing glycerophospho-N-palmitoylethanolamine, γ-linolenic acid, α-tocopherol, monoolein, and fatty acid esters of hydroxy fatty acids. Interestingly, the abundance of *Methylobacterium* differed between *H.pylori*-infected patients and healthy individuals [61].

#### Prevotella

*Prevotella*, a genus of Gram-negative anaerobic bacteria, comprises over 50 species, including *Prevotella melaninogenica* and *Prevotella intermedia*. These bacteria are typically present as commensals inhabiting the human oral cavity, gastrointestinal system, and reproductive organs, but under specific conditions, they may transition into opportunistic pathogens. They are characterized by moderate saccharolytic activity and specific sensitivity to bile salts [62, 63]. Current research has demonstrated that *Prevotella* can contribute to several diseases, including severe asthma [64], atherosclerosis [65, 66], rheumatoid arthritis [67, 68], bacterial vaginosis [69], and human papillomavirus infection-associated cervical lesions [70].

Emerging research has demonstrated the connection between *Prevotella* and the initiation, progression, and prognosis of gastric cancer and its precancerous lesions [71–74]. Unlike *H.pylori*, *Prevotella* has been noted to be more frequently linked to gastric cancer occurring in the upper third of the stomach [75]. Wang *et al.* [24] obtained tissue samples and gastric fluid from clinically healthy individuals, alongside patients with bile reflux gastritis and gastric cancer. Their analysis using 16S rRNA gene sequencing and metagenomics revealed a significant enrichment of melanin-producing bacterial species in the bile reflux gastritis and gastric cancer groups, with *Prevotella melaninogenica* standing out as especially prevalent. Additionally, they observed a significant increase in conjugated bile acids in both groups. Spearman correlation analysis further revealed that four lipopolysaccharide-producing bacteria were significantly positively correlated with elevated levels of conjugated bile acids. To further validate the impact of *Prevotella melaninogenica* and conjugated bile acids on gastric cancer progression, researchers constructed a gastrojejunostomy bile reflux mouse model. The results indicated that *P.melaninogenica* may induce conjugated bile acid, thereby promoting gastric inflammation, precancerous lesions, and the proliferation of gastric epithelial cells mediated by the IL-6/JAK1/STAT3 signaling cascade. Additionally, research has shown that changes in *Prevotella intermedia* associated with gastric cancer can result in decreased expression of fatty acid receptor 2 and short-chain fatty acids, while also driving increased production of inflammatory cytokines such as tumor necrosis factor alpha-induced protein 8 and interleukin 6, and triggering activation of the IL-6/JAK1/STAT3 signaling pathway. This persistent inflammation further elevates the likelihood of developing gastric cancer [76, 77]. Liang *et al.* [78], through microbial culture and next-generation sequencing of gastric cancer tumor tissues, identified an enrichment of *Prevotella intermedia* in the tumor microenvironment. Tumor-derived *Prevotella intermedia* was shown to influence tumor differentiation, perineural invasion, and omental invasion in gastric cancer patients by modulating perilipin 3 protein expression. Additionally, Oosterlinck *et al.* found that certain oral microbial groups, such as *Prevotella*, exhibit high affinity for intestinal mucin-producing tumors, particularly those with Mucin 13 (MUC13) overexpression. Intestinal mucin-producing tumors are often associated with poor prognosis, highlighting the potential role of these oral microbiota as drivers of Mucin 13 signaling, which may accelerate gastric cancer progression [79]. However, other studies have reported higher *Prevotella* abundance in non-atrophic gastritis compared with atrophic gastritis. In this context, *Prevotella* was found to produce acetate, which inhibits nitric oxide secretion and nitric oxide synthase 2 expression in Human Gastric Adenocarcinoma Cells, thereby suppressing gastric cancer development [80].

### Other bacteria

*Lactobacillus*, as a gut probiotic, is generally considered to have anticancer effects. Numerous studies have demonstrated that *Lactobacillus* can alleviate inflammation, reshape the gastric microecological environment in *H.pylori*-infected patients, and produce short-chain fatty acids to inhibit gastric cancer progression [81]. However, some reports suggest that *Lactobacillus* may also promote gastric cancer development through mechanisms such as producing exogenous lactic acid and N-nitroso compounds, inducing oxidative stress, and facilitating epithelial-mesenchymal transition and immune tolerance [82]. The role of other bacteria, such as *Fusobacterium nucleatum*, in gastric cancer is more straightforwardly detrimental. Known for its strong link to colorectal cancer [83], *Fusobacterium nucleatum* has been shown to promote gastric cancer by inducing infected gastric cancer cells to secrete exosomes containing homeobox A1 transcript at the distal tip (HOTTIP). This long non-coding RNA sponges miR-885-3p, leading to the upregulation of EphB2, which in turn activates the PI3K/AKT pathway, promoting tumor cell proliferation and metastasis [84]. Proteins within the MviN domain of *Burkholderia* and *Sphingomonas* have been identified as contributors to natural killer cell activation and the progression of mucosa-associated lymphoid tissue lymphoma [77]. In gastric cancer tissues, *Propionibacterium acnes* is enriched and facilitates macrophage polarization toward the M2 phenotype via the TLR4/PI3K/Akt pathway [85]. Certain pathogenic bacteria, such as *Kytococcus sedentarius*, *Actinomyces oris*, and *Staphylococcus saccharolyticus*, have been shown to promote gastric adenocarcinoma by inducing host DNA methylation [86]. Ling found that genera such as *Stenotrophomonas* and *Selenomonas* are positively correlated with immunosuppressive cells, including BDCA2+ plasmacytoid dendritic cells and T cells, suggesting a potential mechanism for immune evasion by gastric cancer cells [87]. Additionally, *Porphyromonas gingivalis* secretes lipopolysaccharide (LPS), which induces oxidative stress in the gastric mucosa, disrupts the gastric mucosal barrier, and enhances macrophage infiltration. This process increases TNF-α production, which activates TLR2-β-catenin signaling, ultimately promoting gastric cancer development [88].

### Fungi

#### Candida albicans

*Candida albicans* is a common opportunistic pathogenic fungus frequently found on the mucosa of the human gastrointestinal and urogenital tracts [89]. Under normal conditions, *C.albicans* exists in its “yeast form” within the host. However, when homeostasis is disrupted, *C.albicans* undergoes a morphological shift to its “hyphal form,” promoting the onset of various diseases [90]. In daily life, *C.albicans* is often associated with mucosal inflammatory conditions, such as oral thrush and vaginitis [91, 92], as well as respiratory diseases [93, 94], atherosclerosis [95], and cancer [96].

While numerous studies have emphasized the significance of bacteria in gastric tumorigenesis, research on fungi remains limited. *Candida albicans* has been reported to frequently cause cancer-associated gastric ulcers [97]. Advances in high-throughput sequencing have provided further insight into the complex relationship between *C.albicans* and gastric cancer. Through a series of studies, Sano *et al.* [98] revealed that *C.albicans* could trigger chronic inflammation in the stomachs of diabetic mice, resulting in proliferative inflammation of the gastric mucosa, which ultimately developed into gastric cancer. Further research has demonstrated that *C.albicans* can induce type 3 immune responses, thereby affecting the immune microenvironment. Through its hyphal form, *C.albicans* directly damages gastric tissue, triggers epithelial remodeling and signaling pathways, and upregulates genes related to restricted antimicrobial responses, leading to gastric ecological dysbiosis and promoting inflammation in the stomach [99]. Furthermore, Zhong identified a significant abundance of *C.albicans* in gastric cancer tissues relative to adjacent non-cancerous tissues by performing metagenomic sequencing. Their analysis also indicated reduced microbial α and β diversity in cancerous tissues compared with non-cancerous tissues, suggesting that *C.albicans* may promote gastric carcinogenesis by diminishing microbial diversity and altering the gastric microenvironment [26].

### Other fungi

Zhang reported a notable enrichment of *Solicoccozyma* in gastric cancer tumor tissues, with its presence strongly correlated with the Bormann classification and neural invasion in GC patients. Additionally, it was found that *Solicoccozyma* impacts amino acid and carbohydrate metabolism within the tumor microenvironment [100]. As the research advances, it will be crucial to employ more refined methodologies to explore how fungal species modulate tumor biology and engage with the host immune system to promote malignancy. This will offer valuable insights into whether targeting these fungi could provide novel therapeutic strategies for gastric cancer management.

#### Epstein-Barr virus

*Epstein-Barr virus,* or human herpesvirus type 4, is a linear DNA virus capable of spreading through saliva, respiratory droplets, blood, and various other bodily fluids. Once infected, individuals become lifelong carriers of the virus, which may reactivate under conditions of immunosuppression [101]. *EBV* is associated with a variety of diseases, including infectious mononucleosis, Hodgkin’s and non-Hodgkin’s lymphomas, Burkitt’s lymphoma, autoimmune diseases, and epithelial-derived malignancies [101, 102].

**Figure 3:**
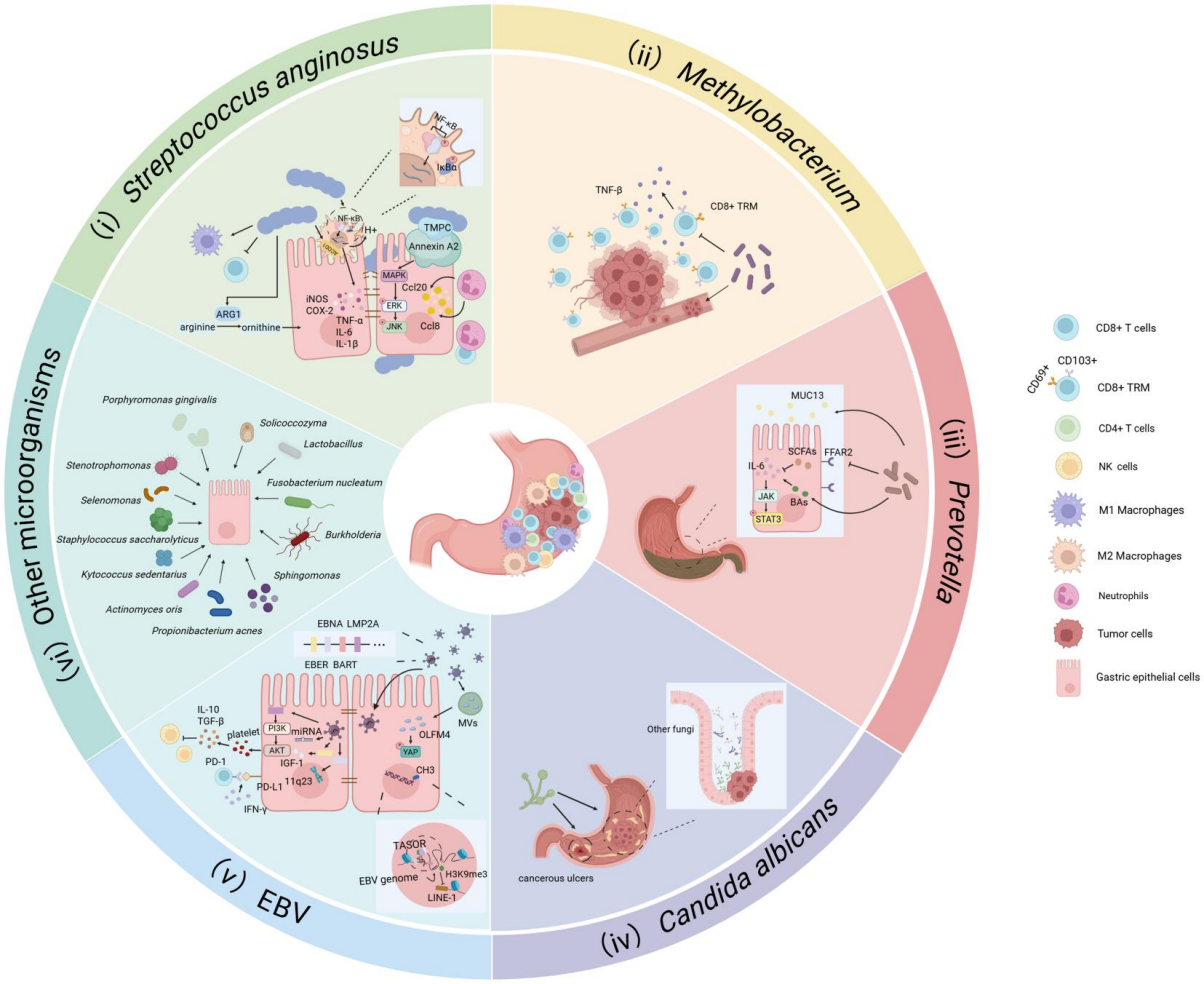
Representative non-*Helicobacter Pylori* pathogenic microorganisms in gastric cancer. (i) *Streptococcus anginosus* disrupts gastric barrier function through the TMPC-ANXA2-MAPK axis, modulating arginine-ornithine metabolism and the tumor microenvironment (e.g. T cells, macrophages, inflammatory mediators, and cytokines), thereby inducing acute and chronic gastric inflammation and promoting gastric tumorigenesis. (ii) *Methylobacterium* suppresses CD103+ CD69+ CD8+ TRM within the tumor microenvironment, reduces TGF-β expression, and facilitates the formation of intravascular tumor thrombi. (iii) *Prevotella* inhibits free fatty acid receptor 2 and SCFAs expression, activates the IL-6/JAK1/STAT3 signaling pathway, promotes gastric inflammation, precancerous lesions, and epithelial cell proliferation, and exhibits a high affinity for intestinal mucin phenotypic tumors, particularly MUC13. (iv) *Candida albicans* reduces microbial diversity and richness in the stomach of gastric cancer patients, predominantly contributing to cancerous ulcer formation. (v) EBV directly invades host cells, induces CpG island methylation phenotypes, encodes viral miRNAs, expresses EBER, EBNA, BARTs, and LMP2A, regulates the cell cycle, enhances PD-L1/PD-1 interactions, upregulates extracellular OLFM4 to activate YAP signaling, and adheres to regions near the TASOR gene-coding area, promoting H3K9me3 deposition on introns and suppressing LINE-1 expression, thereby accelerating gastric tumorigenesis. (vi) Other gastric cancer-associated pathogenic microorganisms. ANXA2, Annexin A2; ACOD1, aconitate decarboxylase; ARG1, Arginase-1; BART, BamHI-A rightward transcripts; BAs, bile acids; Ccl8, C-C motif chemokine ligand 8; Ccl20, C-C motif chemokine ligand 20; COX-2, cyclooxygenase-2; EBER, Epstein-Barr virus encoded RNA; EBNA, Epstein-Barr virus nuclear antigen; EBV, Epstein-Barr virus; ERK, extracellular signal-regulated kinase; FFAR2, fatty acid receptor 2; IGF-1, insulin-like growth factors 1; IL-1β, interleukin 1β; IL-6, interleukin 6; iNOS, inducible nitric oxide synthase; JNK, c-Jun N-terminal kinase; LINE-1, long interspersed element 1; LMP2A, latent membrane protein 2A; LPS, lipopolysaccharide; MAPK, mitogen-activated protein kinase; MUC13, Mucin 13; MVs, microvesicles; NF-κB, nuclear factor kappa-light-chain-enhancer of activated B cells; OLFM4, olfactomedin 4; PD-1, programmed cell death-1; PD-L1, programmed cell death-ligand 1; PI3K, phosphatidylinositol3-kinase; SCFAs, short-chain fatty acids; STAT3, signal transducer and activator of transcription 3; TGF-β, transforming growth factor-beta; TMPC, tripartite multispecificity protein C; TNF-α, tumor necrosis factor-alpha; TRM, tissue-resident memory T. Created with BioRender.com.

Epstein-Barr virus-associated gastric cancer (EBVaGC) represents a unique molecular subtype of gastric cancer that has attracted considerable research interest. Evidence suggests that *EBV* is capable of directly infecting host cells and modulating their gene expression. EBV induces a CpG island methylator phenotype, promoting hypermethylation of host genes, while also inducing hypermethylation of its own genes to facilitate immune evasion, enabling its persistence within the host. Additionally, EBV-encoded microRNAs contribute to processes such as cell migration, invasion, and epithelial-mesenchymal transition [103]. Most EBVaGC cases express Epstein-Barr virus encoded RNA, Epstein-Barr Virus Nuclear Antigen, BamHI-A rightward transcripts, and Latent membrane protein 2A, which regulate the cell cycle. Moreover, the Epstein-Barr virus nuclear antigen 1 protein specifically binds to chromosome 11 in the host, inducing 11q23 fragment breaks and leading to genomic instability, a potential factor underlying cancer susceptibility [103, 104]. Furthermore, Epstein-Barr virus encoded RNA upregulates insulin-like growth factor 1, which accelerates tumor cell proliferation [105]. Duan *et al.* [106] further demonstrated that proteins encoded by *EBV*, such as the latent membrane protein 2A, can trigger the stimulation of the the phosphatidylinositol3-kinase signaling pathway, causing the upregulation of F3 expression. F3-mediated platelet aggregation promotes the release of IL-10 and TGF-β, which suppress NK cell antitumor activity, thereby accelerating gastric cancer progression. Additionally, EBV infection in EBVaGC induces multiple gene mutations, with frequent occurrences of PIK3CA mutations, ARID1A mutations, BCOR mutations, and amplifications of JAK2 and PD-L1 [107]. Another significant factor in EBVaGC is the immune evasion strategy, where EBV infection leads to the overexpression of PD-L1, which interacts with PD-1 to suppress T-cell function, impairing antitumor immunity [108]. Wen *et al.* [109] further identified that EBV-derived microvesicles carry Olfactomedin 4, a gene directly regulated by the cGAS-STING pathway. Upon delivery to recipient cell surfaces by these microvesicles, olfactomedin 4 interacts with the cadherin domain of FAT1, disrupting its intracellular connection with MST1. This disruption leads to the activation of the Hippo-YAP signaling pathway, thereby enhancing cell proliferation. Moreover, other studies have discovered that the EBV genome in EBVaGC patients localizes near the coding region of TASOR, a core protein within the HUSH complex. This enhances interactions between the TASOR enhancer and promoter, significantly upregulating TASOR expression. Elevated TASOR levels promote H3K9me3 deposition on introns, ultimately suppressing LINE-1 expression. This suppression contributes to host genomic instability, further promoting gastric cancer progression [110] (Fig. 3).

## Potential mechanisms of non*-H.pylori* pathogens inducing gastric carcinogenesis

Gastric carcinogenesis is a complex and multifactorial process. While the stomach’s highly acidic environment was once thought to be a barrier to microbial growth, it has become increasingly clear that under certain conditions, exogenous pathogens can infiltrate gastric tissues, and commensal microorganisms from other body sites may translocate into the stomach, resulting in microbial dysbiosis. These pathogens exert their effects on the host through various mechanisms, including direct damage to gastric tissue, long-distance effects, disruption of the gastric microbial ecosystem, and impairment of host immune function [111, 112]. This intricate interplay between host and various microbial pathogens plays a pivotal role in the development of gastric disorders, ultimately leading to gastric cancer. The following outlines the potential mechanisms by which non*-H.pylori* pathogens contribute to gastric carcinogenesis.

### Direct damage to gastric barrier function and invasion of gastric epithelial cells

Throughout infection, pathogens come into contact with various extracellular host proteins, engaging with them in a highly selective way to facilitate processes such as colonization, adhesion, invasion, remodeling of the extracellular matrix, and evasion of the immune system [113]. Some pathogens exert their pathogenic effects by directly engaging with gastric epithelial cells [107]. For instance, *Streptococcus anginosus* employs its surface protein TMPC to interact with the Annexin A2 (ANXA2) receptor present on the surface of gastric epithelial cells, disrupting tight junctions and facilitating persistent adhesion and colonization on the gastric mucosa [21]. Furthermore, mucins, which form the mucus barrier covering gastric epithelial cells, serve as specific binding sites for certain bacteria, promoting chronic inflammation and tumorigenesis [114]. This interaction is particularly relevant for *Neisseria*, *Prevotella*, and *Veillonella*, which exhibit a strong affinity for tumors exhibiting an intestinal mucin phenotype, especially those exhibiting overexpression of Mucin 13. This interaction may represent a mechanism by which these microbes contribute to poor prognoses in gastric cancer [79]. Beyond disrupting gastric barrier function, certain pathogens exert carcinogenic effects by directly invading host cells and modulating host genomic expression, as demonstrated by EBV [103].

### Long-distance effects on gastric carcinogenesis

In addition to directly damaging the gastric mucosal barrier, invading gastric epithelial cells, and influencing host genomic expression, pathogens can also modulate the tumor microenvironment by secreting specific metabolites and extracellular vesicles, thereby regulating tumor growth.

### Influence of metabolite secretion on gastric carcinogenesis

Numerous studies have highlighted the role of pathogenic microorganisms in influencing tumor growth by releasing metabolites like amino acids, bile acids, short-chain fatty acids, inosine, and their associated metabolic byproducts [115]. Yang *et al.* [60], through their analysis of correlations between differential microbiota and metabolites in gastric cancer tissues, found that the abundance of many carcinogenic microbial taxa was positively correlated with pro-carcinogenic metabolites, while negatively correlated with anti-carcinogenic metabolites. Arginine metabolism pathways have been linked to the development of various diseases, with extensive research showing that arginine can be metabolized into polyamines, nitric oxide (NO), and other compounds that regulate the tumor immune microenvironment (e.g. modulating T-cell proliferation and function), activate oncogenic pathways, and ultimately affect cancer progression [116]. *Streptococcus anginosus* exhibits arginase activity, metabolizing arginine into ornithine, which can impact CD8 + T-cell function and thereby promote gastric cancer development and progression [48]. Furthermore, the interplay between microbiota and bile acids is crucial in cancer progression, with the hydrophobicity of bile acids and their ability to generate reactive oxygen species potentially leading to host cell damage, inflammation, and activation of NF-κB. This cascade triggers the secretion of inflammatory cytokines, such as TNF and IL-1β, while inhibiting Farnesoid X Receptor activation, a process that supports tumorigenesis through the Wnt/β-catenin pathway [117]. Additionally, *Prevotella* species may contribute to gastric cancer progression by inducing the production of conjugated bile acids, which activate oncogenic pathways and stimulate gastric epithelial cell proliferation [24]. Moreover, reduced levels of short-chain fatty acids (SCFAs) are often linked to the onset of various diseases, especially those involving butyrate metabolism. SCFAs can inhibit histone deacetylase activity in cancer cells and influence the immune microenvironment by inducing chronic inflammation, promoting CD8+ T-cell exhaustion, and altering dendritic cell IL-27 expression, thereby mediating antitumor effects [118]. For instance, *Prevotella intermedia* has been shown to diminish levels of free fatty acid receptor 2 and SCFAs, thereby elevating the risk of gastric cancer [76, 77]. Additionally, other bacteria, such as *Lactobacillus,* can promote gastric cancer progression by secreting lactate to provide energy for gastric cancer cell growth and by producing carcinogenic compounds such as N-nitroso compounds, which accelerate gastric tumorigenesis [82].

### Influence of extracellular vesicles on gastric carcinogenesis

Extracellular vesicles are biologically active particles of nano-scale dimensions that are produced by cells, capable of carrying a wide variety of bioactive molecules, such as proteins, nucleic acids, and metabolites. These vesicles are crucial for mediating intercellular and microenvironmental signal transduction. Extensive research has demonstrated that EVs mediate interactions between microorganisms and host cells, thereby impacting numerous physiological and pathological processes including immune regulation, inflammatory responses, and nutrient metabolism. These activities can profoundly affect human health and are implicated in the onset and progression of diseases like gastric cancer [119]. Lipopolysaccharide, a major constituent of the outer layer in Gram-negative bacteria, induces the release of pro-inflammatory cytokines through interaction with host cells. This interaction can cause systemic disruptions, potentially leading to conditions like sepsis, endotoxemia, and even tumorigenesis. While free LPS cannot cross the plasma or endosomal membranes to reach the cytoplasm, bacteria can use two primary mechanisms to deliver LPS: direct invasion or secretion via outer membrane vesicles. Outer membrane vesicless are internalized by host cells through endocytosis, transporting LPS into the cytoplasm where it can trigger its biological effects [120]. Studies have reported that host-secreted extracellular vesicles possess an intrinsic ability to bind LPS and transfer it to the cytoplasm via a CD14-dependent pathway [121]. LPS-producing bacteria, such as *Prevotella melaninogenica*, can stimulate gastric epithelial cell growth, thus contributing to gastric cancer development [24]. Similarly, *Escherichia coli* produces LPS that activates the TLR4 receptor and its downstream signaling pathways, including p38, MEK1/2, and TAK1. This activation enhances the adhesion of gastric cancer cells to human peritoneal mesothelial cells, facilitating peritoneal metastasis of gastric cancer [122]. In addition, tumor cells can secrete functional small extracellular vesicles that suppress antitumor immune responses and promote tumor proliferation, invasion, and metastasis. Certain non*-H.pylori* bacteria influence gastric carcinogenesis by modulating the secretion of small extracellular vesicles by tumor cells, either promoting or inhibiting their release [123, 124]. For example, *Fusobacterium nucleatum* has been observed to stimulate infected gastric cancer cells to release exosomes containing HOTTIP. These exosomes can then stimulate the growth and metastasis of uninfected gastric cancer cells [84].

### Disruption of gastric microbial homeostasis

The human microbiota forms a complex and expansive network of microorganisms that play essential roles in health. Pathogen-host interactions often lead to disruptions in the composition and function of the microbiota. For instance, even after the eradication of *H.pylori*, patients often experience persistent microbial dysbiosis, with harmful microbial communities lingering for prolonged periods. This persistent dysbiosis may contribute to the onset and progression of gastric cancer [61]. This highlights the critical role played by these non*-H.pylori* bacteria in subsequent carcinogenic processes. Furthermore, certain pathogens can reduce the abundance of probiotics in the stomach, such as *Faecalibacterium* and *Bifidobacterium*. These probiotics have been shown to produce butyrate, which downregulate PD-L1 and IL-10 expression in immune cells, thereby reversing the immunosuppressive state in gastric cancer patients [112, 125].

### Influence on the gastric immune microenvironment

**Figure 4:**
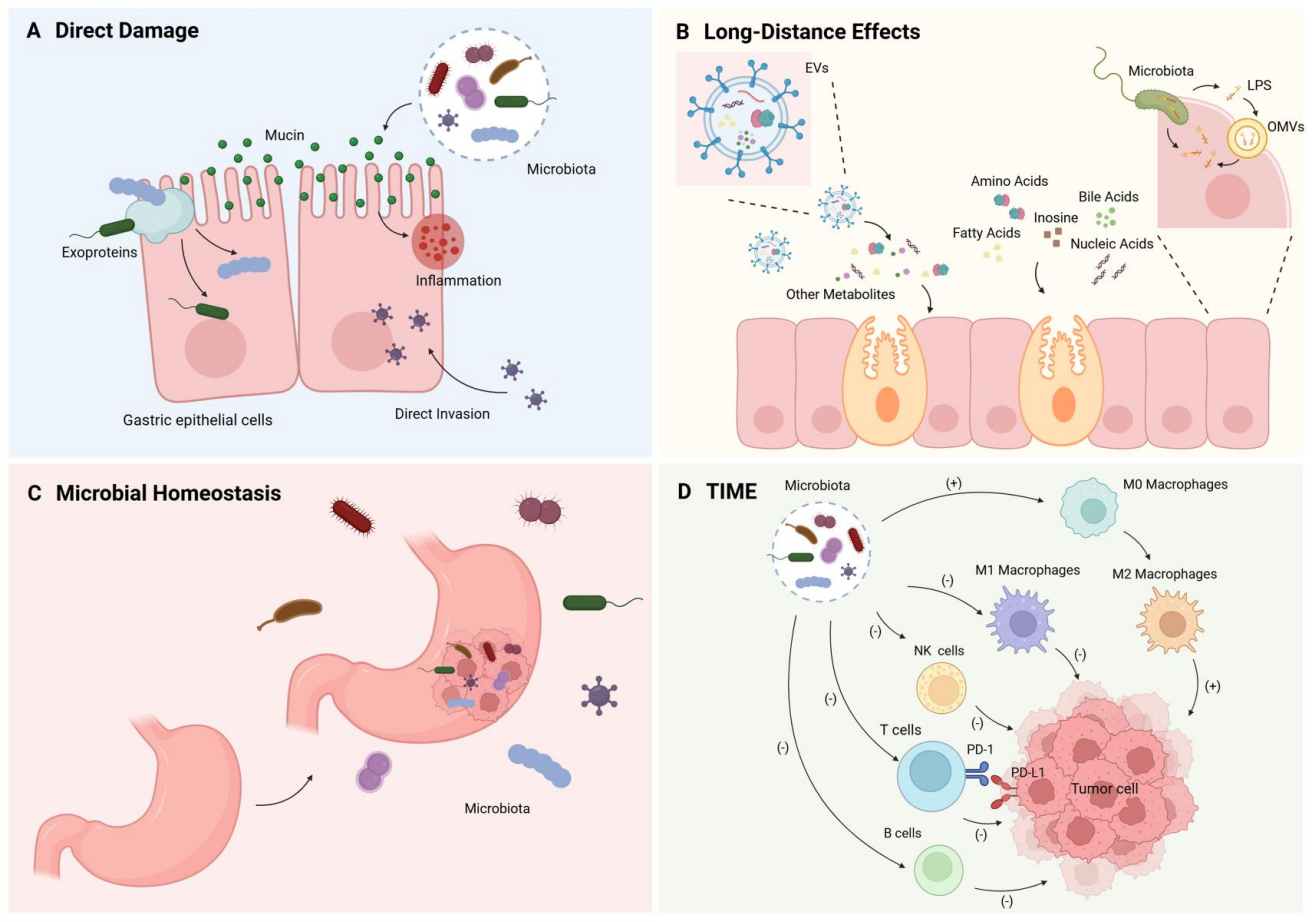
Potential mechanisms of non-*Helicobacter pylori* pathogens inducing gastric carcinogenesis. (A) Direct damage to gastric barrier function and invasion of gastric epithelial cells. (B) Long-distance effects on gastric carcinogenesis. (C) Disruption of gastric microbial homeostasis. (D) Influence on the gastric immune microenvironment. EVs, extracellular vesicles; OMVs, outer membrane vesicles; LPS, lipopolysaccharide. Created with BioRender.com.

The immune microenvironment is pivotal in regulating tumor growth, spread, invasion, and avoidance of immune detection [60]. During tumor development, the immune response can be divided into three distinct phases: immune elimination, immune equilibrium, and immune escape [126]. Pathogens typically exert their pro-carcinogenic effects by disrupting the stability of these phases. The tumor microenvironment consists of tumor cells, various immune cells, fibroblasts, endothelial cells, pericytes, and various other tissue-resident cells, along with blood vessels, the lymphatic system, nerves, and extracellular matrix components [127, 128]. CD8 + T cells are potent cytotoxic effectors in antitumor immune responses and are crucial for eliminating cancer cells [53]. Studies have shown that *Methylobacterium* suppresses tumor-resident memory CD8 + T cells within the tumor microenvironment and reduces TGF-β expression, thereby decreasing their cytotoxicity against gastric cancer cells and promoting immune tolerance [23]. Similarly, *Streptococcus anginosus* has been reported to inhibit CD8 + T cells [37]. In EBV-positive gastric cancer, the interaction between highly expressed PD-L1 and PD-1 further suppresses T-cell proliferation, enhancing immune evasion by gastric cancer cells [55]. Tumor-associated macrophages exhibit considerable plasticity. M1 macrophages are generally linked to pro-inflammatory and antitumor effects, whereas M2 macrophages are associated with tissue repair, immunoregulation, and angiogenesis, which may support tumor growth under certain conditions [53]. *Propionibacterium acnes* has been shown to induce macrophage polarization toward the M2 phenotype, thereby facilitating the growth of gastric cancer cells [56] (Fig. 4).

## Future perspectives in non-*H.pylori* microbiome research

Advances in metagenomics have revealed the gastric microbiome as a complex ecosystem where *H.pylori* represents just one component. This understanding is shifting focus from single-pathogen eradication to ecosystem-level management. The demonstrated role of non-*H.pylori* microorganisms in gastric carcinogenesis warrants developing integrated diagnostic models that incorporate *H.pylori* status, specific non-*H.pylori* taxa abundance, and community structure. Elucidating the molecular mechanisms of these microorganisms will enable the identification of clinical biomarkers and facilitate targeted interventions. Promising approaches include defined probiotics and phage therapies that guide microbial succession toward homeostasis while preventing pathogen dominance. Studies of *H.pylori*-negative gastric cancer further highlight the independent pathogenic potential of non-*H.pylori* communities, suggesting new preventive strategies. Ultimately, understanding non-*H.pylori* microorganisms will establish more comprehensive gastric cancer prevention paradigms, creating new opportunities for precision medicine in oncology.

## Conclusions

Gastric cancer remains a complex, multifactorial disease driven by both host factors and microbial influences. While *H.pylori* has long been recognized as a primary etiological agent, in this review, we have examined the specific mechanisms through which non-*H.pylori* pathogens contribute to the gastric carcinogenesis, highlighting their potential roles in altering gastric microenvironmental factors and influencing tumorigenic processes. These findings underscore the importance of considering a broader spectrum of microbial agents in the pathogenesis of gastric cancer. However, much remains to be understood regarding the synergistic interactions between these microorganisms and their precise roles across different stages of tumorigenesis. Future studies will be crucial in unraveling the cooperative dynamics of these microbes, with the potential to inform new therapeutic strategies targeting microbial communities involved in gastric cancer.

## Authors’ contributions

R.P., G.X., and G.L. supervised the conceptual framework of the manuscript and directed the overall visualization strategy. M.R. and J.J. were primarily responsible for manuscript drafting and figure preparation, and were major contributors in writing the manuscript. Y.L., N.S., and Y.S. assisted in gathering relevant literature materials and conducted language editing and critical revision of the manuscript. Z.W. and X.H. provided expert guidance on the structural design of the figures. All authors read and approved the final version of the manuscript.
